# Pubertal Development Following Paediatric Gonadotoxic Treatment and Immature Testicular Tissue Banking

**DOI:** 10.3390/ijms27052139

**Published:** 2026-02-25

**Authors:** Emily Delgouffe, Marius Regin, Veerle Vloeberghs, Caroline Ernst, Herman Tournaye, Inge Gies, Ellen Goossens

**Affiliations:** 1 Genetics, Reproduction and Development (GRAD) Research Group, Brussels Health Campus/Faculty of Medicine and Pharmacy, Vrije Universiteit Brussel (VUB), Laarbeeklaan 103, 1090 Brussels, Belgium; 2 Department of Gastroenterology, Infectious Diseases and Rheumatology, Charité-Universitätsmedizin Berlin, Hindenburgdamm 30, 12203 Berlin, Germany; 3 Brussels IVF, Universitair Ziekenhuis Brussel (UZ Brussel), Brussels Health Campus, Laarbeeklaan 101, 1090 Brussels, Belgium; 4 Department of Radiology, Division of Paediatric Radiology, Universitair Ziekenhuis Brussel (UZ Brussel), Brussels Health Campus, Laarbeeklaan 101, 1090 Brussels, Belgium; 5 Department of Paediatrics, Division of Paediatric Endocrinology, Universitair Ziekenhuis Brussel (UZ Brussel), Brussels Health Campus, Laarbeeklaan 101, 1090 Brussels, Belgium

**Keywords:** pubertal development, gonadal function, testicular growth, male fertility preservation, immature testicular tissue banking

## Abstract

Paediatric gonadotoxic treatments can compromise male fertility, yet prospective data systematically tracking pubertal development are scarce. Immature testicular tissue banking (TTB) has been introduced as an experimental fertility preservation option for (pre-)pubertal boys, but its long-term safety and interaction with gonadotoxic treatment are not fully understood. This single-centre prospective cohort study systematically followed 23 boys, treated for malignant or non-malignant conditions, between 2017 and 2025 [median 4.0 (0.1–6.9) years], including 15 who underwent TTB. Unlike previous studies, this research combined repeated assessments of pubertal staging, testicular volumes, parenchymal integrity, reproductive hormones, and bone age and density, enabling a multidimensional evaluation of pubertal development. Gonadotoxic treatments, particularly myeloablative conditioning, were associated with reduced post-pubertal testicular volumes and altered hormone profiles, including elevated luteinising hormone and follicle-stimulating hormone, and reduced inhibin B, while anti-Müllerian hormone remained largely stable. Puberty occurred spontaneously and testosterone production was preserved in all patients. The testicular parenchyma appeared unaffected by the biopsy, and although some biopsied testes showed lower volumes, similar reductions could be observed in non-biopsied testes. These results support the safety of TTB, with no evident adverse effects on testicular structure or function; however, larger multicentric prospective studies are needed to confirm these findings.

## 1. Introduction

Each year, approximately 35,000 new cases of childhood cancer are diagnosed in Europe, making cancer the leading cause of disease-related death in children [1]. Advances in cancer treatments have dramatically improved survival rates, with up to 85% of children surviving [2]. With this growing population of childhood cancer survivors being able to live for decades after treatment, it is crucial to understand the long-term side effects of gonadotoxic therapies on the endocrine and gonadal function, as these can lead to disturbed puberty, hormonal imbalances, infertility, and ultimately reduced quality of life. In addition to being used for treating malignancies, gonadotoxic agents are also widely employed as conditioning therapy before haematopoietic stem cell transplantation (CT-HSCT) for non-malignant conditions such as sickle cell disease and bone marrow failure syndromes [3].

Significant clinical evidence indicates a heightened risk of lifelong sterility in boys who undergo such gonadotoxic therapies [4]. However, the exact gonadotoxic mechanism is not completely understood. This is especially true for boys who receive gonadotoxic treatment before or during puberty, a critical developmental period marked by testicular maturation and regulated by changes in hormone levels. In response to this risk, fertility preservation programmes have been developed, offering immature testicular tissue banking (TTB) as an experimental fertility preservation option for (pre-)pubertal boys who cannot benefit from sperm banking. TTB involves the surgical removal and cryopreservation of testicular tissue containing spermatogonial stem cells, with the goal of restoring fertility in adulthood. Three potential restoration strategies are currently being explored: (1) autologous tissue transplantation, (2) isolation and autologous transplantation of the spermatogonial stem cells, and (3) in vitro spermatogenesis. Since its clinical introduction in 2002, the Universitair Ziekenhuis (UZ) Brussel has cryopreserved testicular tissue for over 140 boys, contributing to the more than 3000 boys worldwide who have undergone this experimental procedure. However, despite increasing international uptake, the implementation of TTB remains heterogeneous across centres, and no standardised multicentre protocols have been established [5]. While the testicular biopsy procedure required for TTB is considered low risk, with reported complication rates of only 2–3% [6,7,8], its potential long-term effects on testicular development and function must be considered.

Although standardised, multicentric follow-up studies are lacking, existing single-centred studies have shown that while puberty initiation and testosterone (T) production often remain normal, boys exposed to high-risk gonadotoxic treatments frequently exhibit small adult testicular volumes, elevated serum follicle-stimulating hormone (FSH) levels (indicative of primary testicular failure), and impaired spermatogenesis in adulthood [7,9,10,11,12,13,14,15]. However, most studies were retrospective and have been limited by missing data and small sample sizes, which made it difficult to draw definitive conclusions. Therefore, further comprehensive research is needed to clarify the long-term impact of gonadotoxic therapies and ensure that the biopsy procedure does not exacerbate testicular dysfunction caused by treatment [4].

To address this, the current study employed a prospective follow-up protocol with regular visits over several years, allowing for systematic monitoring of pubertal development in boys treated with presumed gonadotoxic therapies, with or without TTB. These long-term assessments evaluated the impact of gonadotoxic treatments on the gonadal function and explored the potential additional effects of the testicular biopsy procedure. By assessing testicular development, hormonal changes, and pubertal progression at well-defined time points, this research aimed to optimise care for boys with (non-)malignant disorders and refine patient selection criteria for fertility preservation programmes. Our results support the safety of TTB, as no surgical complications or clinically relevant parenchymal abnormalities were observed after biopsy, and continued testicular growth occurred in all patients. Although pubertal progression and T production remained intact, gonadotoxic treatments, particularly myeloablative conditioning, were associated with reduced post-pubertal testicular volumes and impaired reproductive hormone levels.

## 2. Results

### 2.1. Patient Characteristics

**Figure 1:**
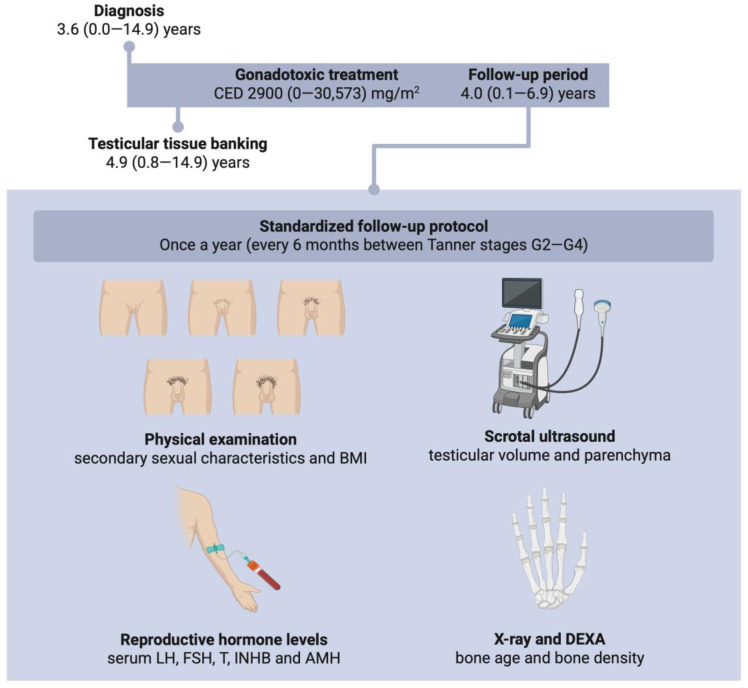
Overview of the study design and follow-up assessments to evaluate pubertal development after paediatric gonadotoxic treatment. This figure outlines the study design and follow-up assessments, which include physical examinations by a fertility specialist, scrotal ultrasounds, reproductive hormone measurements, X-rays, and dual-energy X-ray absorptiometry (DEXA) scans. Created in BioRender. Goossens, E. (2026) https://BioRender.com/4dxrx6v (accessed on 20 February 2026).

A total of 23 patients were included. Their clinical background is summarised in Table 1. At diagnosis, the median age was 3.6 (neonatal-14.9) years (Figure 1), and the patients had received a median cyclophosphamide equivalent dose (CED) of 2900 (0–30,573) mg/m^2^. Of the included patients, twenty were diagnosed with malignant diseases and three with non-malignant diseases. Fourteen patients were treated with conventional high-risk chemo- and/or radiotherapy (HR-C/R) for conditions including Hodgkin lymphoma (4/14), acute lymphoblastic leukaemia (3/14), optic pathway glioma (2/14), Ewing sarcoma (1/14), neuroblastoma (1/14), B cell lymphoma (1/14), Burkitt lymphoma (1/14), and clear cell sarcoma (1/14). The remaining nine patients required CT-HSCT. The diagnoses in this group included acute lymphoblastic leukaemia (2/9), medulloblastoma (2/9), neuroblastoma (1/9), acute promyelocytic leukaemia (1/9), myelodysplastic syndrome (1/9), severe medullary aplasia (1/9), and sickle cell disease (1/9). These patients received various types of conditioning regimens: myeloablative conditioning (MAC) with or without 12 Gy total body irradiation (7/9) or reduced-intensity conditioning (RIC) (2/9). No patients underwent non-myeloablative conditioning. Patient 18, who had sickle cell disease, was treated with hydroxyurea for 4.5 years before undergoing MAC treatment. Patients 7 and 19 also received chemotherapy prior to MAC treatment due to relapse. Patients 21, 22, and 23 received hormone substitution therapy (HST): patient 21 was treated with recombinant human growth hormone (Nutropin; 1.0–1.2 mg/day, subcutaneously) from May 2014 onward; patient 22 received recombinant human growth hormone (Norditropin; 0.5–1.5 mg/day, subcutaneously) from May 2013 to May 2017; and patient 23 was treated with a gonadotropin-releasing hormone analogue (Decapeptyl SR; 11.25 mg intra-muscularly every 10 weeks) from July 2016 to December 2021.

Of the 23 patients, 15 underwent immature TTB at a median age of 4.9 (0.8–14.9) years, typically within two months after their diagnosis. Exceptions were patients with non-malignant haematological disorders. At the time of biopsy, most patients were pre-pubertal (13/15), while one was peri-pubertal and one was post-pubertal. In twelve out of fifteen cases, a hemi-orchiectomy was performed, while two patients underwent a smaller biopsy, and one patient underwent a unilateral orchiectomy. No complications related to the biopsy procedure were recorded. Notably, patients 18 and 19 had already received chemotherapy before TTB (Table 1).

The patients were followed up for a median of 4.0 (0.1–6.9) years. At the final follow-up visit, four patients were pre-pubertal (Tanner stage G1), seven were peri-pubertal (Tanner stages G2–G4), and twelve were post-pubertal (Tanner stage G5). At the time of data collection, some patients were still in follow-up at UZ Brussel (*n* = 7), while for others, follow-up ended either at age 18 (*n* = 11) or after transfer to another centre (*n* = 5). Most patients maintained a normal body mass index (BMI) throughout the follow-up period, except for three patients (patients 1, 7, and 11) who displayed a low BMI for the majority of the follow-up, and three patients (patients 4, 5, and 17) who consistently had a high BMI (Table 2).

Table 2 provides a summary of the scores for BMI, testicular volumes, reproductive hormone levels, bone age, and total bone density at their first and last follow-up visit. To evaluate pubertal development after gonadotoxic childhood treatments, follow-up data were compared between patients treated with a CED < 4000 mg/m^2^ (*n* = 12) and those treated with a CED > 4000 mg/m^2^ (*n* = 8), and between patients who received HR-C/R and those who underwent CT-HSCT (specifically MAC versus RIC). Additionally, to assess the impact of the testicular biopsy procedure, data were further compared between patients who underwent a testicular biopsy and those who did not.

### 2.2. Testicular Development

**Figure 2:**
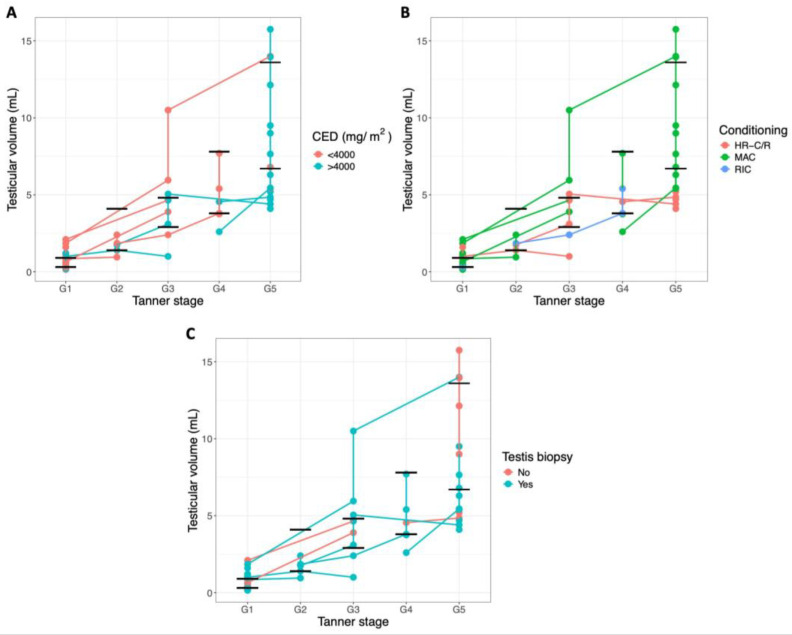
Progression of testicular volumes through pubertal development. Each dot represents the mean testicular volume of an individual patient at a specific follow-up appointment and Tanner stage. Connected dots represent values from the same patient over time. The black lines show the normal reference ranges for testicular volume at each Tanner stage (Table A1). This figure compares testicular growth between patients treated with a CED < 4000 mg/m^2^ and >4000 mg/m^2^ (**A**), between patients who received HR-C/R and CT-HSCT, specifically MAC versus RIC (**B**), and between patients who underwent a testis biopsy and those who did not (**C**). Patients receiving HST (*n* = 3) were excluded from this figure.

**Figure A1:**
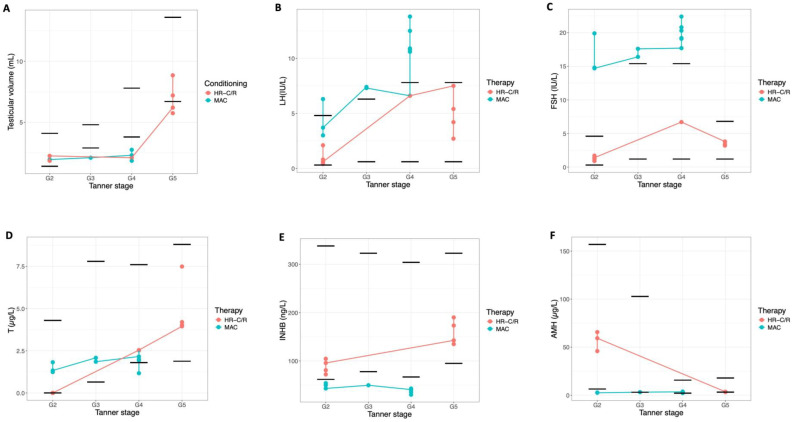
Progression of testicular volumes and reproductive hormone levels through pubertal development of patients receiving HST. Each dot represents the mean testicular volume (**A**) or serum hormone level (**B**–**F**) of an individual patient at a specific follow-up appointment and Tanner stage. Connected dots represent values from the same patient over time. The black lines show the normal reference ranges at each Tanner stage (Table A1). These figures compare the results of patients who received HR-C/R and MAC.

Overall, parenchymal abnormalities on scrotal ultrasound were infrequently observed, with only minor abnormalities detected in 13% of the patients (3/23) during follow-up. In two patients (patients 9 and 20), a mildly lobulated testicular contour was observed in the biopsied testis. In patient 9, this finding was only present at the final follow-up visit at 18 years of age, whereas in patient 20, mild lobulation was observed during puberty but was no longer present post-puberty. In patient 11, several small intra-testicular calcifications were observed in the non-biopsied testis. The progression of testicular growth from Tanner stage G1 to G5 is illustrated in Figure 2. More specifically, testicular volumes were normal in all pre-pubertal patients (9/9). During early puberty (Tanner stages G2–G3), 57% of patients (4/7) already exhibited volumes below the reference range. The frequency of abnormalities increased further during the later Tanner stages (G4–G5), with 70% of patients (7/10) showing reduced testicular volumes (patients 3, 5, 6, 9, 11, 19, and 20). This was especially evident in the patients who underwent CT-HSCT (MAC and RIC), who consistently exhibited low-normal testicular volumes during pubertal development. Notably, all patients who underwent MAC exhibited abnormally low testicular volumes at Tanner stage G5, while no clear differences in testicular volumes were observed between patients with a CED < 4000 mg/m^2^ and those with a CED > 4000 mg/m^2^. Patients 21–23, who received HST, also exhibited reduced testicular volumes at Tanner stage G5, consistent with these observations (Figure A1). Although all patients showed testicular growth regardless of the biopsy procedure (Table 2), reduced post-pubertal testicular volumes were observed in both biopsied and non-biopsied patients. This finding was more pronounced in the biopsied group, where reduced volumes were observed either bilaterally or confined to the biopsied testis.

### 2.3. Reproductive Hormone Levels

**Figure 3:**
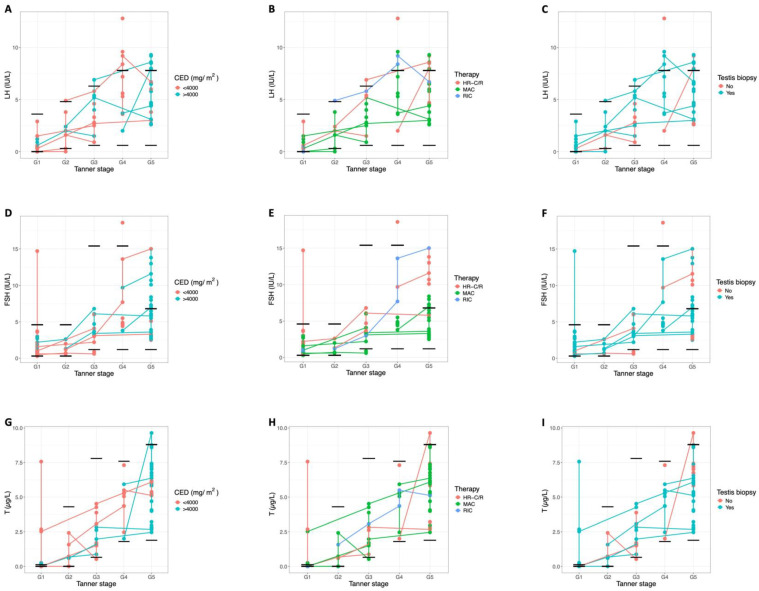

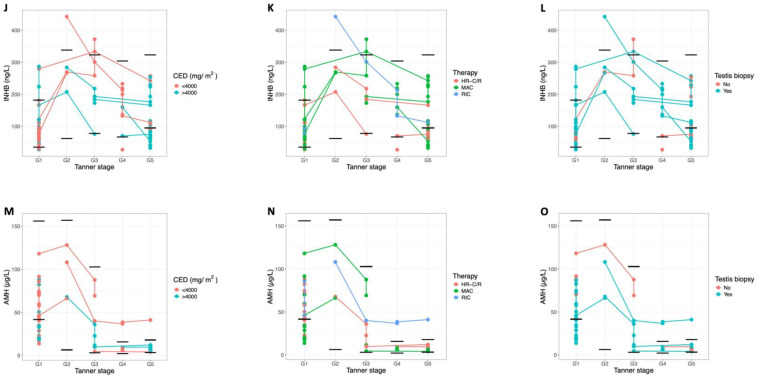
Progression of reproductive hormone levels through pubertal development. Each dot represents the serum hormone level of an individual patient at a specific follow-up appointment and Tanner stage. Connected dots represent values from the same patient over time. The black lines show the normal reference ranges for testicular volume at each Tanner stage (Table A1). This figure compares the levels of LH (**A**–**C**), FSH (**D**–**F**), T (**G**–**I**), INHB (**J**–**L**), and AMH (**M**–**O**): between patients treated with a CED < 4000 mg/m^2^ and >4000 mg/m^2^ (**A**,**D**,**G**,**J**,**M**), between patients who received conventional HR-C/R versus CT-HSCT, specifically comparing MAC versus RIC (**B**,**E**,**H**,**K**,**N**), and between patients who underwent a testicular tissue biopsy and those who did not (**C**,**F**,**I**,**L**,**O**). Patients receiving HST (*n* = 3) were excluded from this figure.

The evolution of the reproductive serum hormone levels through puberty is shown in Figure 3. For most patients, serum gonadotropin levels remained within the normal range pre-puberty (9/9) and during Tanner stages G2 (4/5) and G3 (6/7). However, as with testicular volumes, most hormonal disturbances were observed during the later Tanner stages (G4–G5), where 42% of the patients (5/12) exhibited elevated luteinising hormone (LH) levels (patients 3, 7, 18, 19, and 20) and 42% (5/12) showed elevated FSH levels (patients 3, 7, 11, 18, and 20). Patients who underwent CT-HSCT (MAC or RIC) consistently demonstrated elevated LH and FSH levels during the later Tanner stages. T levels remained normal for almost all patients throughout follow-up, and none required T replacement therapy during puberty. Only a few patients (patients 8, 12, and 14) showed transiently elevated T at the end of their G1 visits, likely reflecting the onset of G2. Importantly, all T levels normalised during the remainder of follow-up. While almost all patients maintained normal inhibin B (INHB) levels before (8/9) and during puberty (9/11), half of the patients (5/10) exhibited low INHB levels post-puberty (patients 3, 5, 9, 11, and 18). Low anti-Müllerian hormone (AMH) levels could be observed before puberty in 25% of the patients (2/8) (patients 10 and 12), whereas they were normal for all patients (6/6) during the early Tanner stages (G2–G3) and for all but one patient (9/10) (patient 20) during the later Tanner stages (G4–G5). Patients 21–23, who received HST, were analysed separately: patient 21, who also underwent MAC-based CT-HSCT with total body irradiation, showed multiple hormonal abnormalities at Tanner stage G5, including elevated LH and FSH and low T, INHB, and AMH, while patients 22 and 23, who did not receive MAC, maintained largely normal hormone levels (Figure A1). Overall, no clear differences in hormone levels were observed between patients treated with a CED < 4000 mg/m^2^ and those with a CED > 4000 mg/m^2^, nor between biopsied and non-biopsied patients. However, there was a noticeable trend toward lower post-pubertal INHB levels in patients who received a higher CED.

### 2.4. Bone Age and Bone Density

Bone age abnormalities were observed in 37% (7/19) of the patients, with five (patients 4, 5, 9, 17, and 19) showing an advanced bone age and two (patients 6 and 20) exhibiting a delayed bone age. Among these, three patients had undergone CT-HSCT (two received MAC and one RIC). No notable differences in bone age were identified between patients treated with a CED < 4000 mg/m^2^ and those treated with a CED > 4000 mg/m^2^. All patients with an abnormal bone age had undergone a testicular biopsy. Among the HST patients, bone age was normal in patients 21 and 22, while patient 23 showed advanced bone age, consistent with prior treatment with gonadotropin-releasing hormone analogues for central precocious puberty. Bone density measurements were largely within the normal range for most patients throughout the follow-up period. However, specific abnormalities were identified in a small subset: 2/16 patients showed a heightened trabecular bone density (patient 12: treated with radiotherapy, and patient 16: treated with RIC), 1/17 exhibited a reduced lumbar bone density (patient 11: treated with radiotherapy), and 1/16 displayed a reduced total bone density (patient 19: treated with MAC). All four patients had undergone a testicular biopsy, and three of them (excluding the patient treated with RIC) had received a CED of >6500 mg/m^2^. Bone density was normal in all three patients receiving HST.

## 3. Discussion

Many boys who undergo gonadotoxic therapies, whether for cancer or non-malignant conditions, face significant risks concerning their pubertal development, gonadal function and future fertility. In response to this, the fertility preservation strategy of immature TTB has been introduced, offering hope for boys who cannot yet benefit from sperm banking. However, the exact long-term effects of these gonadotoxic treatments, along with the potential additional risks associated with the biopsy required for TTB, remain unclear. This uncertainty makes it challenging to determine which patients should be considered for TTB, highlighting the need for more comprehensive research. To address this gap in our knowledge, the current study followed a cohort of 23 patients who received paediatric chemo- and/or radiotherapy, including some undergoing a biopsy for TTB. Over several years, the study systematically monitored their pubertal progression and testicular development, aiming to provide deeper insights into the long-term effects of gonadotoxic therapies and a testicular biopsy for fertility preservation.

Our findings confirm that obtaining a testicular biopsy is safe in the short term [6,7,8], as no post-surgical complications were reported in our cohort. Minor parenchymal abnormalities were observed in three patients. Two patients showed mild lobulation in the biopsied testis: in one patient, the finding was transient and limited to puberty, while in the other, it was observed only at the final visit. It is unclear whether these changes are related to the biopsy or represent incidental morphological variation. In another patient, small calcifications could be observed in the non-biopsied testis, clearly unrelated to the biopsy procedure. Overall, these subtle abnormalities were limited in extent and did not show progressive changes over time. Most importantly, consistent with earlier studies, continued growth of the biopsied testis was observed, reinforcing the safety of the procedure for fertility preservation [7,10,11,14,15,16]. While testicular volumes were generally normal before puberty, many patients exhibited reduced volumes post-puberty. This reduction was particularly noticeable in biopsied patients and patients treated with MAC-based CT-HSCT protocols. These findings align with earlier studies [10,17], including our retrospective analysis [15] that suggest that individuals treated with MAC-based CT-HSCT protocols are particularly at risk for impaired testicular development. The pronounced effect after MAC likely reflects the vulnerability of the germ cell compartment to intensive conditioning (high cumulative doses of alkylating agents and/or radiotherapy), causing profound spermatogonial depletion and, consequently, restricted tubular expansion during puberty [18]. Interestingly, recent evidence demonstrates that childhood cancer survivors treated with chemotherapy alone generally displayed recovery to age-appropriate testicular volumes in adulthood. In contrast, exposure to testicular radiation ≥ 1 Gy (median dose, 12 Gy) resulted in persistently low testicular volumes without late catch-up growth, underscoring the long-term suppressive effect of even low-dose testicular irradiation on testicular development [19].

In line with these observations, the hormone profiles also displayed a clear age-dependent shift: although values were generally normal before and during early puberty, elevations in LH and/or FSH became increasingly common in the later Tanner stages, occurring in almost half of the patients. These elevated gonadotropin levels suggest potential gonadal impairment across affected patients, with a particularly pronounced effect in the patients who were treated with CT-HSCT. This pattern is consistent with primary hypogonadism, as intensive gonadotoxic treatment depletes the germ-cell population and disrupts the testicular niche, reducing testicular feedback and thereby triggering a compensatory rise in gonadotropins [18,20].

Nevertheless, T levels remained sufficiently high across all patients throughout the follow-up period, and no patients required T replacement therapy. A few patients showed transient elevations at the transition from G1 to G2, likely reflecting the onset of puberty, but all levels normalised subsequently. While all patients experienced spontaneous puberty, abnormalities in bone age were observed, with 26% showing advanced bone age and 11% showing delayed bone age. These deviations may reflect the impact of gonadotoxic treatment on pubertal progression and skeletal maturation. However, individual variability in pubertal timing cannot be excluded, as information on parental pubertal onset was not available. Half of the post-pubertal patients exhibited low INHB levels, indicative of a disrupted Sertoli cell function [21]. A trend toward lower INHB levels was observed in patients who received a higher CED. Interestingly, some pre-pubertal patients also exhibited reduced AMH levels, which may serve as an early sign of gonadal dysfunction [22].

These results align with previous studies on long-term gonadal outcomes after HR-C/R or CT-HSCT which also reported elevated gonadotropin levels, low INHB levels, and low-to-normal T levels post-puberty [9,10,11,12,13,15,17,23]. Our retrospective study demonstrated that patients treated with MAC-based CT-HSCT showed significantly more cases of low INHB levels compared to those treated with HR-C/R (41% vs. 5%) [15]. The detrimental effects of CT-HSCT are further emphasised by the findings of Mathiesen et al. who reported that 23% of patients treated with CT-HSCT required T substitution to induce puberty, and 27% of those who underwent spontaneous puberty developed T deficiency in early adulthood [12]. Although chemotherapy-induced Leydig cell failure leading to androgen insufficiency and the need for T substitution therapy is relatively uncommon [18,24], Mathiesen et al. demonstrated that higher cumulative testicular irradiation doses significantly increased the risk of T substitution and gonadal dysfunction [13]. Additionally, in patients who experienced spontaneous puberty, the bone age was often significantly delayed [12]. Similarly, the study of Borgström et al. that also exclusively focused on CT-HSCT patients, found that 79% exhibited advanced puberty, and while T levels remained normal during follow-up, 40% of patients eventually required T substitution treatment in adulthood [10], underscoring the importance of long-term follow-up into adulthood to fully assess the impact of treatment. Although the reported rates of endocrine dysfunction and T substitution vary between cohorts, the overall pattern across studies is consistent, with spontaneous pubertal onset in most patients but a higher risk of persistent Sertoli cell impairment and late Leydig cell insufficiency after intensive CT-HSCT regimens. Differences in cohort composition, cumulative radiation exposure and duration of follow-up likely account for the variability in severity reported in the literature.

These hormonal findings have important implications for fertility restoration strategies after immature TTB. Elevated post-pubertal gonadotrophins and low INHB levels point toward underlying testicular dysfunction, particularly impaired Sertoli cell function [20,21,22]. Because testicular tissue is collected prior to gonadotoxic therapy, the immature germ and somatic cells are expected to remain structurally and functionally intact. In the context of testicular tissue auto-transplantation, high FSH levels stimulate Sertoli cell proliferation and maturation, while high LH levels induce Leydig cell maturation and subsequent androgen production, thereby creating a favourable environment for graft development and functionality [25]. Together with the presence of adequate T levels in adulthood, these conditions could support the ability of transplanted tissue to mature and initiate spermatogenesis. In contrast, spermatogonial stem cell auto-transplantation relies on the endogenous testicular environment for engraftment and differentiation. After gonadotoxic treatment, recipient Sertoli cells may be compromised, limiting their ability to provide a supportive niche for transplanted germ cells and thereby reducing the likelihood of success. Post-pubertal hormonal imbalances should therefore be considered a potential barrier for successful stem cell auto-transplantation outcomes.

An important limitation of our study is the relatively small cohort size, including a considerable number of patients who have not yet completed puberty. As most gonadal dysfunctions are anticipated to manifest during and after puberty, a longer follow-up period that spans the entire pubertal timeline is essential. Ideally, patients should be monitored from before puberty until adulthood to fully understand the long-term effects of gonadotoxic treatments. Furthermore, although no hormonal differences were observed between biopsied and non-biopsied patients in our cohort, the study was not powered to formally assess the impact of the biopsy procedure on testicular function. Due to the above-described limitations, we did not carry out logistic regression analysis and analysed our data only from a descriptive point of view. Therefore, no definitive conclusions regarding a potential additional effect of biopsy can be drawn, and larger cohorts with a higher number of non-biopsied patients are needed to address this question more robustly.

Finally, our findings need to be interpreted within the broader context of fertility preservation outcomes. There are still no reported human pregnancies or live births from cryopreserved testicular tissue, and clinical translation in this area remains experimental. Even ovarian tissue cryopreservation, despite being clinically implemented for more than two decades, has resulted in relatively few births, with a recent systematic review identifying 170 newborns after autologous transplantation [26]. This highlights the importance of continued long-term follow-up in cohorts with stored testicular tissue.

## 4. Materials and Methods

The fertility preservation programme at UZ Brussel has been active since 2002; however, the structured prospective follow-up protocol forming the basis of this study was only introduced in 2017. Consequently, the prospective follow-up of patients after childhood gonadotoxic treatment was conducted between November 2017 and December 2025 according to the protocol detailed below.

### 4.1. Patients

This single-centre prospective cohort study included young boys (<18 years old) who underwent gonadotoxic treatments during childhood for malignant or non-malignant conditions and were eligible for immature TTB due to the high infertility risk associated with their treatment protocols. At the time of diagnosis, these patients were referred to the fertility preservation programme at UZ Brussel by paediatric oncologists and haematologists from UZ Brussel and Hôpital Universitaire des Enfants Reine Fabiola (HUDERF). Both those who accepted and those who declined the TTB procedure were invited to participate in this study. Exclusion criteria were a diagnosis of testicular cancer and disease relapse.

### 4.2. Interventions

#### 4.2.1. Intake

During oncological or haematological intake, the patient’s (non-)malignant condition and the planned gonadotoxic treatment protocol were discussed with the patient and his parents. Detailed treatment data were systematically recorded to enable accurate assessment of the individual and combined effects of various therapeutic modalities. This included documentation of the chemotherapeutic agents administered, the site, type, and dose of radiotherapy, the use of total body irradiation, the type and frequency of surgical interventions, and the specifics of conditioning regimens prior to HSCT. The intensity of the chemotherapy was determined by calculating the CED. A CED value exceeding 4000 mg/m^2^ was used as the threshold for further data analysis to identify treatments associated with a significant risk of infertility, based on previous research [27,28].

#### 4.2.2. Fertility Preservation

Following oncological or haematological intake, experienced fertility specialists or nurses provided information to the patient and their parents about the risk of lifelong sterility associated with the patient’s condition and/or planned treatment. Fertility preservation via immature TTB was proposed to the patient and their parents. After obtaining written informed consent from the parents and/or the patient (if aged 12 years or older), testicular tissue was collected by a urologist through an open surgical procedure under general anaesthesia. This procedure was often combined with central line placement for chemotherapy administration or bone marrow aspiration prior to HSCT. Based on testicular volume at the time of biopsy and in agreement with the patient and/or their parents, either a unilateral orchiectomy, hemi-orchiectomy, or a small biopsy (removal of 10–25% of the testis) was performed. The largest testis was selected for surgery. When both testes were equal in size, the left testis was selected because it is generally easier to access during surgery and may offer slightly more favourable venous drainage, potentially reducing the risk of venous congestion after surgical manipulation. For hemi-orchiectomies and small biopsies, tissue was collected from the lower pole. The excised testicular tissue was divided into smaller fragments (1–20 mm^3^) and cryopreserved using a standardised slow-freezing protocol [29]. The patient’s age at the time of the procedure, along with the side and size of the testicular biopsy, was documented. Clinical assessment of pubertal maturation, including Tanner staging [30], was conducted, together with a measurement of the testicular volumes. Additional family history, such as maternal age at menarche, paternal pubertal age, sibling pubertal timing, and any fertility-related issues, were also recorded to support the evaluation of pubertal development.

#### 4.2.3. Follow-Up Assessments

Follow-up visits occurred at least annually from the completion of treatment until adulthood. During puberty (Tanner stages G2–4), the visit frequency was increased to every six months to allow closer monitoring of pubertal progression. Clinical evaluations were conducted by a small team of experienced endocrinologists and included anthropometric measurements (height, weight, and BMI), blood pressure assessment under resting conditions, and evaluation of secondary sexual characteristics using Tanner staging. Z-scores for the patient’s BMI were calculated according to the Belgian growth references (https://mngsbe.github.io/zscore.be, accessed on 1 December 2025), with values ranging from −2.0 to 2.0 considered within the normal range. Information on HST, including treatment type, dosage, and start and end dates, was also collected.

Testicular volumes and parenchymal abnormalities, such as fibrotic lesions and calcifications, were assessed via scrotal ultrasound performed by a radiologist using a 14L5 linear transducer (Canon Medical Systems N.V., Zaventem, Belgium). Testicular volumes were calculated using the formula: length × width × height × 0.52 [31] and compared to the reference values per Tanner stage (Table A1) [32].

Blood samples were collected to evaluate gonadal function through the measurement of reproductive hormones, including LH, FSH, T, INHB, and AMH. Hormonal determinations were performed by the Clinical Chemistry & Radioimmunology laboratory of the UZ Brussel using the Elecsys LH, FSH, T, and AMH assays on the Cobas e immunoassay analyser (Roche Diagnostics, Machelen, Belgium) or the INHB Gen II enzyme-linked immunosorbent assay kit (Diagnostics Systems Laboratories, Webster, TX, USA). The reference values per Tanner stage are summarised in Table A1.

Bone age was assessed annually using X-rays of the hand and wrist and evaluated according to the Greulich and Pyle atlas. This provided an estimate of pubertal maturation due to the correlation between skeletal maturation and the hypothalamic–pituitary–gonadal axis [33]. The bone age was scored via the Bayley–Pinneau method and was considered normal if it deviated by no more than one year from the patient’s actual age. To monitor bone quality in cases of potential T deficiency, trabecular spine-, lumbar spine-, and total body bone mineral density were measured annually using DEXA scans [34]. Z-scores were calculated for each measurement, with values between −2.0 and 2.0 considered normal.

### 4.3. Statistical Analysis

Follow-up data analysis and descriptive statistics were performed using MS Excel version 16.74 (Microsoft, Redmond, WA, USA) and RStudio version 2023.06.0+421 (Posit Software, PBC, Boston, MA, USA). Data are presented as median (range) unless stated otherwise. For each patient, all measurements (testicular volume, hormone levels, bone age, and bone density) were scored as normal or abnormal (low/high) at each follow-up visit. To summarise values per Tanner stage, the most frequently observed score within that stage was assigned to the patient. In cases where a patient had equal numbers of normal and abnormal measurements within a Tanner stage, the stage was classified as abnormal. Missing data were not imputed; measurements that were unavailable at a given visit were simply left blank and excluded from descriptive summaries. Patients receiving HST were included in the patient characteristics section; however, their follow-up data were analysed separately and excluded from figures and percentage calculations, as exogenous hormone exposure may influence pubertal development.

## 5. Conclusions

Taken together, our findings suggest that paediatric gonadotoxic treatments, particularly MAC-based CT-HSCT, are associated with reductions in post-pubertal testicular volumes and alterations in reproductive hormone profiles, including elevated LH and FSH levels and reduced INHB. AMH levels remained largely stable, with reductions observed only in a subset of pre-pubertal patients. Despite these changes, pubertal progression occurred spontaneously in all patients, and T production remained preserved. Importantly, the testicular parenchyma appeared unaffected by the testicular tissue biopsy. While some biopsied patients exhibited lower post-pubertal testicular volumes, similar reductions were also observed in non-biopsied patients, and no other clear detrimental effects of the biopsy were evident. Overall, these findings support the feasibility of performing immature TTB in pre-pubertal patients without obvious adverse effects on pubertal development. However, given our small sample size and heterogeneity in diagnosis and treatment regimens, statistical confirmation will require larger multicentric cohorts. Future studies should not only expand sample size but also extend follow-up into adulthood and harmonise follow-up procedures across centres, allowing more accurate evaluation of long-term gonadal outcomes and clearer identification of which patients may benefit most from immature TTB.

## Figures and Tables

**Table 1:** Clinical background of the patients.

Patient Number	Number of Visits	Disease	Age at Diagnosis (Years)	Testicular Tissue Banking	Age at Banking (Years)	Treatment Type	Gonadotoxic Treatments
**1**	9	Medulloblastoma	3.9	Hemi-orchiectomy left	3.9	MAC	MET-HIT 2000-BIS4 protocol: chemotherapy CED 11,956 mg/m^2^ and 324 mg/m^2^ cisplatin
**2**	6	Neuroblastoma	0.2	No	-	HR-C/R	SIOP 2001 Nephroblastoma/Infant Neuroblastoma protocol: chemotherapy CED 2900 mg/m^2^ and 937 mg/m^2^ carboplatin
**3**	5	Neuroblastoma	2.0	No	-	MAC	SIOP HR-NBL protocol: chemotherapy CED 4129 mg/m^2^, and 158 mg/m^2^ cisplatin, 1452 mg/m^2^ carboplatin and radiotherapy: 21 Gy right adrenal lodge, 15 Gy vertebral column, 21 Gy left parietal skull
**4**	6	B cell lymphoma	3.6	Hemi-orchiectomy, side N/R	3.6	HR-C/R	B-NHL HR protocol: chemotherapy CED 3777 mg/m^2^
**5**	3	Acute lymphoblastic leukaemia	13.1	Hemi-orchiectomy right	13.1	HR-C/R	EsPhALL VHR protocol: chemotherapy CED 6455 mg/m^2^
**6**	6	Hodgkin lymphoma	14.9	Smaller biopsy right	14.9	HR-C/R	OEPA/COPDAC protocol: chemotherapy CED 2103 mg/m^2^
**7**	1	Acute promyelocytic leukaemia	4.0; relapse at 6.9	No	-	MAC	AIDA/relapse APL protocol: chemotherapy CED 0 mg/m^2^ and radiotherapy: 12 Gy total body irradiation
**8**	7	Medulloblastoma	2.9	Hemi-orchiectomy left	3.0	MAC	SFCE/ACNS 0334 protocol: chemotherapy CED 0 mg/m^2^ and 2400 mg/m^2^ carboplatin and local radiotherapy: 50.4 Gy
**9**	3	Hodgkin lymphoma	9.9	Hemi-orchiectomy left	10.0	HR-C/R	Euronet PHL C1 protocol: chemotherapy CED 1066 mg/m^2^
**10**	6	Acute lymphoblastic leukaemia	0.8	Hemi-orchiectomy left	0.8	HR-C/R	Interfant 06 protocol: chemotherapy CED 1395 mg/m^2^
**11**	9	Ewing sarcoma	3.2	Smaller biopsy right	3.2	HR-C/R	EURO-EWING 99 protocol: chemotherapy CED 30,573 mg/m^2^ and radiotherapy: 54 Gy anterior of the femur
**12**	8	Clear cell sarcoma	1.4	Hemi-orchiectomy right	1.5	HR-C/R	SIOP 2001 protocol: chemotherapy CED 6500 mg/m^2^ and radiotherapy: 10 Gy
**13**	5	Acute lymphoblastic leukaemia	3.3	No	-	HR-C/R	EORTC 58081 AR1 protocol: chemotherapy CED 3266 mg/m^2^
**14**	6	Hodgkin lymphoma	8.1	Hemi-orchiectomy right	8.1	HR-C/R	Euronet PHL C1 protocol: chemotherapy CED 2632 mg/m^2^
**15**	4	Burkitt lymphoma	6.9	No	-	HR-C/R	UKCCSG 2003 B protocol: chemotherapy CED 4432 mg/m^2^
**16**	4	Myelodysplastic syndrome	0.8	Hemi-orchiectomy right	0.8	RIC	Personalised conditioning protocol: chemotherapy CED 0 mg/m^2^
**17**	5	Hodgkin lymphoma	5.0; relapse at 8.6	Hemi-orchiectomy left	5.0	HR-C/R	Euronet PHL C1/relapse protocol: chemotherapy CED 18,984 mg/m^2^ and 11,454 mg/m^2^ cisplatin
**18**	2	Sickle cell disease	0.0	Orchiectomy right	6.0	MAC	Bu/Cy conditioning protocol: chemotherapy CED 8860 mg/m^2^ and 4.5 years hydroxyurea before banking
**19**	5	Acute lymphoblastic leukaemia	2.1; relapse at 4.6	Hemi-orchiectomy right	4.9	MAC	EORTC 58081 protocol before banking/IntReALL HR protocol: chemotherapy CED 3997 mg/m^2^
**20**	5	Severe medullary aplasia	7.9	Hemi-orchiectomy right	8.3	2 × RIC	Personalised conditioning protocol: chemotherapy CED 15,500 mg/m^2^ and radiotherapy: 2 Gy total body irradiation
**21 (HST)**	24	Acute lymphoblastic leukaemia	3.0	No	-	MAC	EORTC 58951 VHR protocol: chemotherapy CED 1527 mg/m^2^ and radiotherapy: 12 Gy total body irradiation
**22 (HST)**	1	Optic pathway glioma	5.5	No	-	HR-C/R	SIOP-LGG 2004 protocol: chemotherapy CED 1539 mg/m^2^ and 64 mg/m^2^ cisplatin and radiotherapy: 50.4 Gy
**23 (HST)**	13	Optic pathway glioma	0.8	No	-	HR-C/R	SIOP-LGG 2001 protocol: chemotherapy CED 0 mg/m^2^ and 7196 mg/m^2^ carboplatin

Abbreviations: N/R, not recorded.

**Table 2:** Testicular volumes, reproductive hormone levels, bone age, and total bone density at the first and last follow-up visit.

Patient Number	Age (Years)	Tanner Stage	BMI (kg/m^2^)/ Z-Score	Testicular Volume Left/Right (mL)	LH (IU/L)	FSH (IU/L)	T (µg/L)	INHB (ng/L)	AMH (µg/L)	Bone Age (Years)	Total Bone Density (mg/mL)/Z-Score
**1**	8.0	G1	**13.1/−2.3**	0.3 */**0.2**	<0.3	0.6	<0.1	**27.6**	50.4	8.0	259.0/−1.0
	14.0	G3	**14.4/−2.3**	1.2 */0.8	1.5	6.8	0.9	**76.1**	-	13.5	297.9/0.1
**2**	12.4	G1	16.9/−0.4	2.3/1.9	<0.3	0.6	<0.1	122.2	118.0	13.0	-
	14.7	G3	-	-	3.3	**0.9**	3.9	**371.8**	69.2	-	-
**3**	14.4	G4	**15.4/−2.1**	6.0/**3.1**	2.0	9.7	2.0	70.3	9.4	14.0	-
	17.5	G5	18.0/−1.5	-	**9.2**	**15.0**	6.7	**70.9**	9.7	-	-
**4**	8.6	G1	**28.7/2.8**	1.2/0.9	<0.3	0.9	<0.1	90.4	59.7	**10.0**	243.3/−1.5
	13.6	G2	**32.1/2.3**	3.3/1.5	3.8	2.0	0.7	267.1	66.3	14.0	265.5/−0.8
**5**	16.7	G5	-	7.3/5.3 *	6.6	**7.3**	3.9	116.7	7.9	**18.0**	-
	17.8	G5	**30.9/2.3**	-	6.5	5.9	4.7	**83.6**	6.3	-	-
**6**	15.7	G4	17.3/−1.4	4.0/3.5 *	3.6	4.6	2.5	136.7	9.2	-	282.3/−0.9
	17.6	G4	-	-	5.6	4.9	5.3	232.9	6.3	-	-
**7**	18.0	G4	**16/−3.0**	-	**12.8**	**18.6**	7.3	**27.1**	-	-	-
	-	-	-	-	-	-	-	-	-	-	-
**8**	8.0	G1	15.4/−0.3	**0.2** */**0.2**	<0.3	1.6	<0.1	62.5	42.8	**6.0–7.0**	252.0/−1.4
	13.1	G1	18.8/0.2	1.6 */1.6	2.9	**14.7**	2.7	167.5	40.4	13.0	264.7/−0.8
**9**	16.0	G5	20.0/0.1	4.8 */6.0	3.8	6.0	5.4	**89.3**	5.0	**18.0**	299.7/−0.8
	18.1	G5	-	5.0 */8.6	-	-	-	-	-	-	-
**10**	4.9	G1	15.6/0.1	**0.2** */**0.2**	<0.3	0.3	<0.1	**29.8**	**13.7**	-	-
	9.0	G1	17.1/0.5	-	<0.3	0.7	<0.1	89.5	**28.6**	-	-
**11**	13.5	G4	**14.2/−2.7**	3.8/1.4 *	3.7	3.8	5.9	159.7	-	13.0–13.5	-
	17.6	G5	**17.1/−2.0**	15.0/4.0 *	**9.3**	6.5	8.7	**38.1**	-	-	276.3/−1.9
**12**	5.3	G1	-	**0.2**/**0.2** *	<0.3	0.7	<0.1	-	-	-	-
	10.8	G1	14.1/−1.8	1.7/0.6 *	0.9	2.7	**0.2**	**223.7**	**17.9**	11.0	275.8/−0.4
**13**	8.6	G1	15.6/−0.3	0.4/0.4	<0.3	0.8	<0.1	58.0	70.1	9.0	283.0/−0.3
	13.9	G3	21.0/0.9	3.4/4.3	4.6	6.0	1.7	-	-	13.0	284.1/−0.2
**14**	13.7	G1	18.6/0.0	1.6/2.1 *	1.5	1.6	**2.5**	**279.1**	-	**11.5**	263.1/−0.6
	17.7	G5	20.2/−0.3	20.2/7.8 *	6.0	3.3	6.2	257.4	5.3	-	-
**15**	14.3	G5	19.3/0.1	8.0/10.0	2.6	2.6	7.1	223.8	5.1	15.0	251.1/−1.2
	17.5	G5	22.1/0.5	16.0/15.5	4.7	2.8	7.2	193.0	5.0	18.0	-
**16**	4.1	G1	-	0.3/0.3 *	0.4	1.1	<0.1	95.1	45.9	-	-
	6.7	G1	-	-	<0.3	0.9	<0.1	-	-	-	252.1/−1.4
**17**	12.3	G1	**27.7/2.1**	-	**4.0**	3.2	**1.5**	172.8	-	-	-
	16.1	G5	-	-	**8.6**	**8.4**	3.0	229.7	-	-	-
**18**	16.7	G5	27.3/1.7	9.4/0.0 *	4.7	**13.0**	2.8	**84.4**	12.0	17.0–18.0	304.2/−1.1
	17.7	G5	28.4/1.9	8.2/0.0 *	**8.5**	**13.8**	3.2	**61.3**	9.6	18.0	-
**19**	11.4	G2	23.1/1.5	2.4/1.1 *	2.4	1.3	0.6	284.0	67.9	**13.0**	**214.4/−2.1**
	15.4	G5	27.4/1.8	6.3/2.5 *	**8.6**	5.8	2.7	166.1	12.1	**17.0**	293.7/−1.0
**20**	14.0	G1	19.8/0.1	3.2/0.5 *	**4.9**	1.2	**1.6**	**442.3**	108.0	**11.5–12.5**	-
	17.9	G5	-	-	6.7	**15.0**	5.1	112.3	**41.1**	**16.0–17.0**	-
**21 (HST)**	13.4 ^#^	G2	22.9/1.2	1.7/2.2	**6.3**	**19.9**	1.8	**50.0**	**2.8**	13.0	-
	18.2 ^#^	G5	-	**2.6/2.9**	**12.5**	**19.1**	**1.2**	**35.7**	**2.2**	-	**186.7/−2.0**
**22 (HST)**	17.0	G5	20.6/0.0	**6.0/5.5**	5.4	3.8	7.5	135.0	-	-	313.5/−1.0
	-	-	-	-	-	-	-	-	-	-	-
**23 (HST)**	9.9 ^#^	G2	23.0/1.7	1.6/2.1	2.1	1.7	<0.1	72.2	65.7	**11.5**	263.1/−0.6
	15.5	G5	24.9/1.4	**5.8**/11.9	4.2	3.4	4.0	190.1	-	**17.0**	197.1/−0.5

Abnormal scores are indicated in bold. The volume of the biopsied testis is indicated with *. Follow-up assessments performed during hormone substitution therapy are indicated with ^#^.

## Data Availability

The dataset supporting the results of this study is securely stored in the Vrije Universiteit Brussel (VUB) Institutional Data Repository under restricted access, with the accession number VUB/BITE/1/000004, to protect participant privacy. Access requests will be reviewed on an individual basis and must be directed to Prof. Ellen Goossens (ellen.goossens@vub.be). She will assess the request, considering the research purpose and potential commercial applications. Prior to any data sharing or release, a data use agreement, in compliance with the VUB legal department’s guidelines, must be completed and signed. Metadata for the dataset is available through the VUB Research Portal at https://researchportal.vub.be/en/datasets/prospective-patient-follow-up-18-years (accessed on 20 February 2026).

## Abbreviations

The following abbreviations are used in this manuscript:

GRAD	Genetics, Reproduction and Development
VUB	Vrije Universiteit Brussel
UZ	Universitair Ziekenhuis
TTB	Testicular tissue banking
CT-HSCT	Conditioning therapy before haematopoietic stem cell transplantation
T	Testosterone
FSH	Follicle-stimulating hormone
CED	Cyclophosphamide equivalent dose
HR-C/R	High-risk chemo- and/or radiotherapy
MAC	Myeloablative conditioning
RIC	Reduced intensity conditioning
HST	Hormone substitution treatment
N/R	Not recorded
DEXA	Dual-energy X-ray absorptiometry
BMI	Body mass index
LH	Luteinizing hormone
INHB	Inhibin B
AMH	Anti-Müllerian hormone
HUDERF	Hôpital Universitaire des Enfants Reine Fabiola

## Appendix A

**Table A1:** Reference values for testicular volumes and hormone levels per Tanner stage.

	Tanner Stage				
	G1	G2	G3	G4	G5
**Testicular volume** (mL)	0.3–0.9	1.4–4.1	2.9–4.7	3.8–7.8	6.7–13.6
**LH** (IU/L)	0.0–3.6	0.3–4.8	0.6–6.3	0.6–7.8	0.6–7.8
**FSH** (IU/L)	0.3–4.6	0.3–4.6	1.2–15.4	1.2–15.4	1.5–6.8
**T** (µg/L)	<0.1	<0.1–4.3	0.6–7.8	1.8–7.6	1.9–8.8
**INHB** (ng/L)	35.0–182.0	62.0–338.0	78.0–323.0	67.0–304.0	95.0–323.0
**AMH** (µg/L)	41.6–155.9	6.4–156.9	3.1–102.8	2.1–15.7	3.2–17.9

Detection limits: 0.05 IU/L for LH, 0.30 IU/L for FSH, 0.025 μg/L for T, 2.91 ng/L for INHB, and 0.16 μg/L for AMH. Data were retrieved from https://www.scribd.com/document/489110645/Pediatric-Reference-Ranges-Endocrinology-0981-pdf (accessed on 3 September 2025).
